# Mechanical and Thermal Characterization of Bamboo and Interlaminar Hybrid Bamboo/Synthetic Fibre-Reinforced Epoxy Composites

**DOI:** 10.3390/ma17081777

**Published:** 2024-04-12

**Authors:** Matilde Oliveira, Vitor Neves, Mariana D. Banea

**Affiliations:** 1 DEMaC—Department of Materials and Ceramic Engineering, University of Aveiro, 3810-193 Aveiro, Portugal; matilde.c.oliveira@ua.pt; 2 NewStamp—Estampagem de Componentes Metalicos, Lda., Rua da Paz, nº 113-115, Cacia, 3800-587 Aveiro, Portugal; 3 CICECO—Aveiro Institute of Materials, Department of Materials and Ceramic Engineering, University of Aveiro, 3810-193 Aveiro, Portugal

**Keywords:** composite materials, hybrid composites, natural fibres, synthetic fibres, bamboo fibre

## Abstract

The main objective of this study was to investigate the mechanical and thermal properties of bamboo, as well as interlaminar hybrid composites reinforced with both bamboo and synthetic fibres in an epoxy matrix. Bamboo and glass, aramid, and carbon bidirectional fabrics were used with a bi-component epoxy matrix to fabricate the composite materials using the vacuum bagging process. The synthetic fabrics were placed on the outer layers, while the bamboo fabrics were used as the core of the hybrid composites. The developed composites were characterized and compared in terms of morphological, physical, and mechanical properties. Further, thermogravimetric (TGA) analysis was used to measure and compare the degradation temperature of the composites studied. Finally, a Scanning Electron Microscopy (SEM) analysis was performed in order to examine the fracture surfaces of the specimens tested. It was found that the fibre hybridization technique significantly improved the general mechanical properties. TGA analysis showed an increase in the thermal stability of the composites obtained by incorporating the synthetic fibres, confirming the effect of hybridization and efficient fibre matrix interfacial adhesion. The results from this work showed that the use of synthetic fibre reinforcements can help to significantly improve the mechanical and thermal properties of bamboo fibre-reinforced composites.

## 1. Introduction

Given the environmental constraints imposed by conventional materials, the scientific community has actively pursued a shift towards greener alternatives across diverse industrial sectors. The use of such materials would improve ecological impacts stemming from carbon dioxide (CO_2_) emissions into the atmosphere, not only during extraction but also in usage and disposal phases. This would significantly diminish the carbon footprint linked with currently utilized materials, such as synthetic composites, over their entire lifecycle [1]. A potential solution involves promoting the use of more sustainable materials, such as natural fibre-reinforced composites, as an alternative to the currently used synthetic reinforcements (carbon and glass fibres, among others) [2].

Natural fibres as sustainable natural materials (e.g., jute, sisal, flax, hemp, curauá, ramie, bamboo, among others) have increasingly become the focus of scientific studies [3,4]. Generally, these fibres are lighter and more cost-effective, and the associated CO_2_ emissions in both their production and disposal are comparatively lower compared to the synthetic fibres [5]. Furthermore, the plants from which these fibres are extracted produce oxygen for the environment and absorb carbon dioxide, contributing positively to environmental sustainability [6]. Thus, natural fibre constitutes an important class of reinforcements utilized in fibre-reinforced polymer composites. However, natural fibres have some associated disadvantages, including water absorption, rapid degradation, property volatility, and low resistance to high temperatures [7,8].

Bamboo fibres are well known for their strength, stiffness, distinctive microfibrillar angle with the fibre axis, and thicker cell wall and are considered as “nature’s glass fibre” [9]. For these reasons, bamboo fibre gained significant interest as a sustainable reinforcement fibre in polymer composites [10,11]. As the use of natural fibre as reinforcement has proliferated in the automotive industry due to legislation driving energy-efficient vehicles made of lightweight, biodegradable, and recyclable materials [2,12], bamboo fibre-reinforced composites are considered a promising natural fibre reinforcement for automotive applications [13].

Several researchers studied the mechanical behaviour of bamboo fibre-reinforced polymer composites [14,15,16,17]. It was shown in the literature that the combination of more than two types of fibre (natural and synthetic fibre) can increase the mechanical and thermal properties of composites [3]. Additionally, it can reduce the water absorption on the natural fibre-reinforced composite materials [17]. The effect of hybridization on the performance of bamboo fibre-reinforced composites was investigated [18,19,20,21,22,23,24,25,26,27,28], with most of the literature focusing on bamboo/glass [24,25,26] and bamboo/carbon fibre hybridization [23,27]. For instance, Mandal et al. [25], investigated bamboo/glass hybrid composites and found that bamboo fibres can replace up to 25 wt.% of glass fibres without lowering the mechanical properties of glass fibre-based composites. Vijaya Kumar et al. [26] studied the hybridization of bamboo composites with glass fibres with different staking sequences. The results revealed that composite samples of G/B/G/G (45% glass fibre, 15% bamboo fibre, and 40% epoxy resin) offered stronger mechanical strength than the other three hybrid samples. Samal et al. [18] found that adding glass fibre enhanced the mechanical (both flexural and impact) and thermal properties of bamboo and glass fibre-reinforced polypropylene hybrid composites. Raghavendra Rao et al. [28] investigated the effect of hybridization of bamboo with glass fibre on flexural and compressive properties of composites and also found that the flexural and compressive properties improved after the incorporation of glass fibre.

In a recent work, Kore et al. [27] studied the mechanical behaviour of bamboo composites hybridized with carbon fibres. They state that the characteristics of hybrid composites can be customized to yield automotive components that are not only desirable in terms of performance but are also cost-effective and sustainable. However, a comprehensive analysis of the effect of synthetic fibre hybridization in the bamboo composite material properties for application in the automotive industry remains little explored in the existing literature. The objective of this research was to investigate the mechanical and thermal properties of bamboo and interlaminar hybrid bamboo/synthetic fibre-reinforced epoxy composites. Bamboo and glass, aramid, and carbon bidirectional fabrics were used with a bi-component epoxy matrix to fabricate the composite materials using the vacuum bagging process. The synthetic fabrics were placed on the outer layers, while the bamboo fabrics were used as the core of the hybrid composites. The developed composites were characterized and compared in terms of mechanical, physical, and morphological properties. Further, TGA analysis was used to measure and compare the degradation temperature of the composites studied. Finally, a SEM analysis was performed in order to examine the fracture surfaces of the specimens tested.

## 2. Materials and Methods

Neat bamboo fibre-reinforced composites and interlaminar hybrid bamboo/synthetic fibre (glass, aramid, and carbon) composites were fabricated using bamboo and bidirectional glass/aramid/carbon fibre fabrics. The bamboo fabrics were supplied by Cobratex (Carbonne, France), and the bidirectional carbon and aramid fabrics were supplied by Rebelco (Alcabideche, Portugal), while the glass fabrics were supplied by Jushi (Tongxiang, China). The basic parameters of the fabrics used are summarized in Table 1 (data provided by the suppliers). The natural fibres did not receive any surface treatments. A commercially available epoxy resin, commonly used in industry to produce composites, was selected as matrix. A bi-component epoxy resin, SR8100, was used in combination with a SD8824 hardener with a weight ratio of 100:22, supplied by Sicomin Epoxy Systems (Châteauneuf-les-Martigues, France).

### 2.1. Specimen Manufacturing

**Figure 1:**
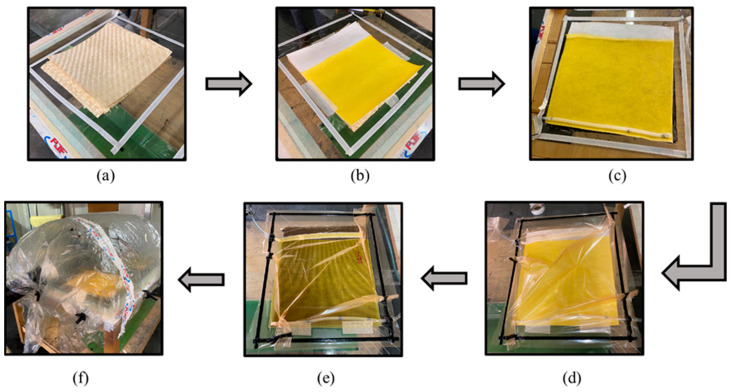
Composite specimen manufacturing process: (**a**) hand lay-up of bamboo fibres; (**b**) placement of flow mesh on top of fibres; (**c**) resin inlet tube insertion; (**d**) vacuum bag placement; (**e**) infusion process; (**f**) post curing process.

The composites (the neat bamboo fibre-reinforced composites and interlaminar hybrid bamboo/synthetic fibre (glass, aramid, and carbon)) were fabricated using the vacuum bagging technique (schematically illustrated in Figure 1). The manufacturing process was initiated with the cutting of the fabrics to the desired dimensions (350 by 350 mm as can be seen in Figure 1a). After this step, a mould release wax was applied to the glass surface of the table used for the composite processing. A pre-drying process of the bamboo fabrics was carried out, as recommended by the supplier, to ensure the absence of moisture in the fibres, which could impact the resin polymerization process and consequently compromise the mechanical properties of the final composites. After this step, the mass of the fibres was subsequently measured for each case and the composite material was hand laid up (see Figure 1a). Following this, the mixing of the epoxy resin SR 8100 with the hardener SD 8824, with a 100:22 ratio between the resin and hardener, was carried out, and further, the vacuum system was activated. The process was carried out until the complete wetting of the fabrics occurred (Figure 1e). Following this, the curing process was initiated according to the resin supplier’s recommendations (Figure 1f). Finally, the plates were cut into specimens with dimensions specified in the ASTM standards for each test, D3039/D3039M-14 (tensile tests), ASTM D7264/D7264M-15 (flexural tests), and ASTM D4812-11 (impact tests), using the water jet cutting technique. For the tensile specimens, the dimensions of specimens were 250 mm × 25 mm in length and width, and for the flexural specimens, they were 125 mm × 13 mm, while for the impact specimens, they were 64 mm × 13 mm. The thickness of the specimens was approx. 4 mm for the pure bamboo specimens, approx. 5 mm for the hybrid composites, and approx. 2 mm for the pure glass fibre-reinforced composites.

The stacking sequence was such that a nucleus of 5 bamboo layers was enveloped by 2 layers of synthetic fabrics (glass, carbon, and aramid) on each side of the specimens. This configuration was chosen based on the literature and the previous experience of the authors (it was found in the literature to be more advantageous to place the synthetic layers on the outside of the natural fibre core) [29,30,31].

**Figure 2:**
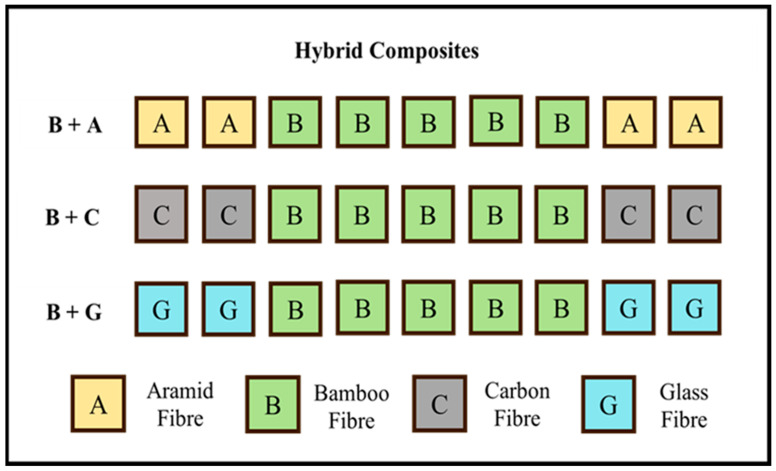
Schematic of stacking sequence of fibres in hybrid composites.

The fibre weight percentage of fibres in the hybrid composites was kept at around 40% and the resin + hardener (100:22) at 60% of the final composite weight. The weight ratio of bamboo to synthetic fibre in the hybrid B + A, B + C, and B + G composites was approx. 75/25, 80/20, and 60/40%, respectively. Schematics and the nomenclature of the specimens according to the material and reinforcement are depicted in Figure 2.

### 2.2. Measurements and Characterization

#### 2.2.1. Morphological Analysis

For the fibre analysis, a scanning electron microscopy (SEM) analysis was performed using the Analytical UHR Schotty emission scanning electron microscope SU-70 (Tokyo, Japan), with a voltage of 8 kV. The measurement of the fibre diameter was carried out using Image J software (1.54d version). Five measurements were taken for each fibre type, and an average was calculated based on these measurements.

For the examination of fracture sections of the composites (previously subjected to tensile tests), a SEM analysis was performed using the low-vacuum SEM equipment TM4000Plus Hitachi (Tokyo, Japan), operating at a voltage of 10 kV. All studied samples underwent a gold–palladium deposition using the K950X Turbo Evaporator Emitech (Montigny le Bretonneux, France) prior to analysis.

#### 2.2.2. Density Characterization

Two distinct methods, the geometric method (Equation (1)) and the Archimedes’ method (Equation (2)), were used for the density characterization:

(1) $$\rho =\frac{m}{V}$$

(2) $$\rho_{apparent}=\frac{M_{D}}{M_{W}-M_{I}}\times \rho_{liquid}$$

where *ρ* is the density in g/cm^3^, *m* is the mass in g, *V* is the volume in cm^3^, *M_D_* is the mass of the dry specimens, *M_W_* is the mass of the wet specimens, and *M_I_* is the mass of the immersed specimens.

The apparent density of the composites was obtained by following the principle of Archimedes (Equation (2)) and recommendations of ASTM C830-00 using a precision balance (sensitivity 0.1 mg). First, each sample was dried in a stove overnight, and the dried sample mass, *M_D_*, was weighed immediately after removing from the stove. Each sample was then immersed in water for 3 h (*M_I_* parameter was measured), and the saturated weight of samples, *M_W_*, was weighed in air subsequently.

#### 2.2.3. Mechanical Analysis

**Figure 3:**
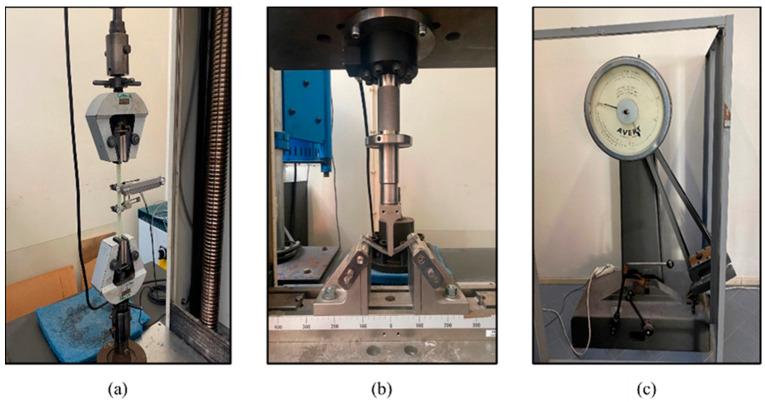
Example of the composites test set-up: (**a**) tensile; (**b**) flexural; (**c**) impact.

The specimens were tested with the aid of a Shimadzu AG-25 TA universal testing machine (Kyoto, Japan), see Figure 3a,b. For the tensile tests, a 20 kN load cell was used for the bamboo fibre composites, and a 100 kN load cell was used to test the synthetic fibre and hybrid composites. A 105 mm three-point bending rig was used for the flexural tests (see Figure 3b). The flexural stress and strain were calculated as per the international standard ASTM D 7264 via Equations (3) and (4).

(3) $$\sigma =\frac{3PL}{2bh^{2}}$$

(4) $$\varepsilon =\frac{6\delta h}{L^{2}}$$

The impact tests were conducted using the Charpy Leeds LS10 2DE equipment (Birmingham, England, UK) as can be seen in Figure 3c. At least four specimens were tested for each condition. All tests were conducted at room temperature.

#### 2.2.4. Thermal Analysis

TGA was performed in a NETZSCH TG 209 F3 Tarsus machine (Netzsch-Gerätebau GmbH, Wiesbaden, Germany). Samples of approximately 20–25 mg were used to make the measurements. An alumina (Al_2_O_3_) crucible was used. Each sample was tested in the temperature range of 30 °C–600 °C at a constant heating rate of 10 °C/min under nitrogen atmosphere (20 mL min^−1^).

## 3. Results and Discussion

### 3.1. Morphological Analysis

#### Fibre Analysis

**Figure 4:**
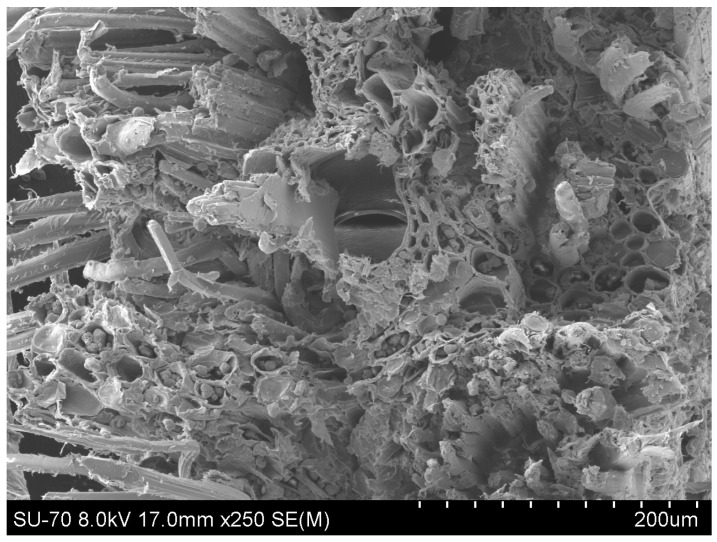
Cross-sectional SEM image of bamboo fibre.

Firstly, the cross-sectional area of the bamboo fibre was analysed to understand the structural differences between synthetic and natural fibres. Figure 4 shows the image obtained in the SEM analysis, and it can be seen that in the bamboo fibre, elements typical of plant cells, such as the cell wall and the lumen, are clearly identifiable, similar to other studies from the literature [10,32,33].

**Figure 5:**
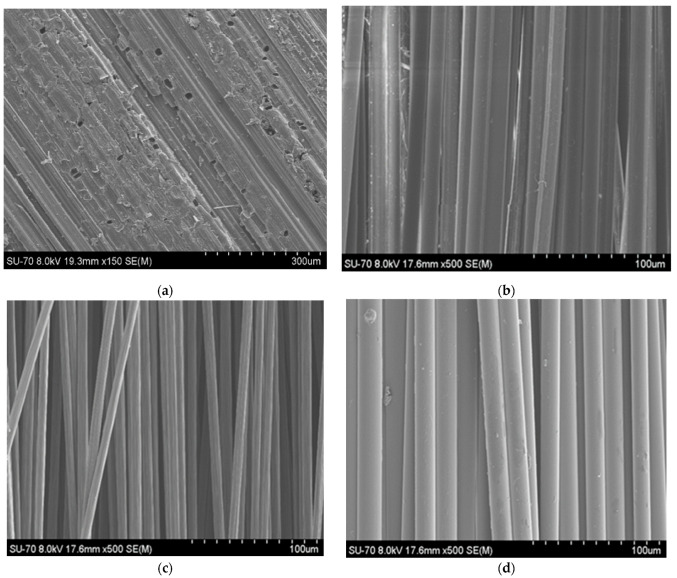
Representative SEM images: (**a**) bamboo fibre; (**b**) aramid fibre; (**c**) carbon fibre; (**d**) Glass fibre.

Figure 5 presents representative SEM images of the fibres used in this study. The diameter of each individual fibre bundle was determined using the Image J software. Five measurements were taken for each fibre type, and an average was calculated based on these measurements. The diameters of the fibre bundles were measured to be 80.17 ± 9.02 µm for the bamboo fibres. Takeuchi et al. [34] measured bamboo fibre diameters and found diameter values varying from 250 μm to 500 μm. They obtained the bamboo fibre from both the outer and inner region in bamboo strips by hand and showed that the fibres with smaller diameters in both outer and inner regions presented higher tensile strength.

The diameters determined for carbon and aramid fibres were 21.3 ± 2.14 µm and 32.5 ± 2.80 µm, respectively, while for the glass fibre, it was 41.0 ± 0.94 µm. It was shown in the literature that the diameter and length of fibres have an impact on the mechanical characteristics of composites [3]. In general, longer and thinner fibres exhibit superior mechanical properties due to improved fibre–matrix bonding and enhanced load transmission. Therefore, it is crucial to take into account the physical attributes of fibres when developing polymeric composites for specific applications.

### 3.2. Density Characterization

Table 2 presents the quantitative density data and shows that the values for geometric and apparent densities are similar across all cases studied, with the latter being slightly higher than the former.

The B batch of specimens presented the lowest density values as expected, while for the hybrid composites, the density increased according to the fibre type used for the hybridization. Given that the same resin was used for all composites, it was expected that the density of the hybrid composite specimens would follow the same trend as the densities of the fibres used. Similar results were found in the literature for B composites [16]. Latha et al. [35] determined the density of bamboo fibre-reinforced composites with epoxy matrix and obtained a value of 1.11 g/cm^3^ for a composite with four layers of bidirectional bamboo fabrics, while for bamboo/glass hybrid fibre-reinforced composites, a value of 1.208 g/cm^3^ was found.

### 3.3. Mechanical Characterization

#### Tensile Properties

**Figure 6:**
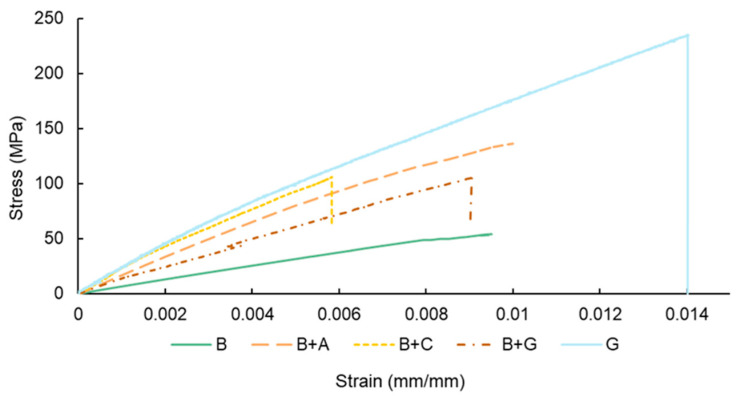
Representative tensile stress–strain curves as a function of hybridization.

**Figure 7:**
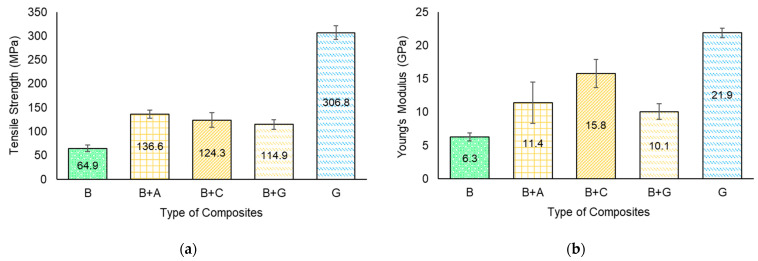
Tensile material properties as a function of hybridization architecture, (**a**) tensile strength; (**b**) Young’s modulus.

Figure 6 shows the representative tensile stress–strain curves, while Figure 7 and Table 3 present the quantitative tensile properties of the composites as a function of hybridization.

The tensile strength values varied as a function of fibre reinforcement material. The lowest values were found for the B specimens (64.9 MPa). The tensile strength of bamboo fibre-reinforced composites varied according to the type and size of the bamboo fibres used, the resin utilized as the matrix, and the manufacturing technique used to produce the composite, which limited the percentage of fibres. For instance, Sudarisman et al. [36] found a tensile strength and elastic modulus of approx. 80 MPa and 6.4 GPa, respectively, for bamboo–epoxy composites. The difference might be explained by the different manufacturing technique used to manufacture the composites (i.e., compression moulding).

**Figure 8:**
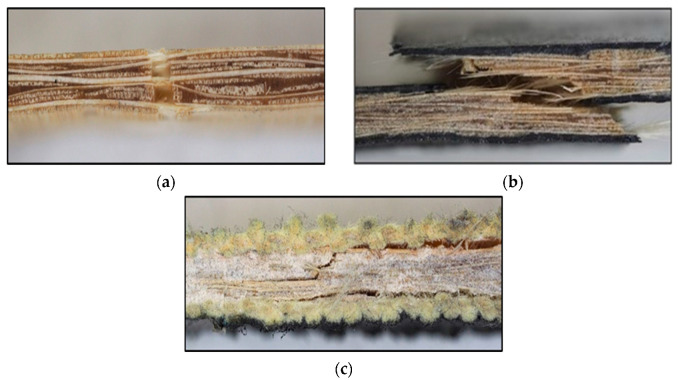
Representative tensile specimen failure mode: (**a**) B; (**b**) B + C; (**c**) B + A.

For the hybrid bamboo composites, the synthetic outer layer significantly increased the tensile strength of the materials due to the high strength of the synthetic fibre used. For example, the B + C specimens presented an enhancement in tensile strength of approx. 92% when compared to the B specimens, while the B + G presented an improvement of approx. 77% (see Figure 7a). A higher variation was observed for the B + A specimens, with an improvement in tensile strength of approx. 111%. The higher values obtained for batch B + A can be explained mainly by the fact that the content of aramid fibres within the hybrid composite was higher in batch B + A (25% vs. 20% of carbon fibre in B + C batch) and by the failure mode of the B + A specimens (illustrated in Figure 8c).

The hybridization of bamboo fibre-reinforced composites with different synthetic fibres also resulted in a significant increase in stiffness when compared to the batch reinforced solely with bamboo fibres. The largest increase in the stiffness parameter was recorded for the B + C specimens (approx. 150%), followed by batch B + A (approx. 81%), and lastly, batch B + G (approx. 60%).

From Figure 8, representative tensile failures can be seen for the B, B + C, and B + A specimens (the other groups (B + G) presented similar failures to B + C specimens). From Figure 8a, the partial failure of the B specimen is visible. Resin matrix cracking occurs on both sides of the specimen, debonding completely from the core. The core itself does not rupture completely for all specimens. This is linked to the difference in ductility between the fibre and matrix, where the less ductile phase will rupture first at a lower strain.

On the other hand, from Figure 8b, the failure of the B + C case can be seen, and the total failure of the specimen is observed. This difference from the neat B case is likely due to the fact that the C fibres have a more brittle tendency and fail quickly at lower strains, transferring all the load to the bamboo core/resin, which suffer a load transfer similar to an impact, leading to a total rupture. From Figure 8c, the representative failure cross-section of the B + A specimens can be seen. Differently from the C fibres, the A fibres are highly ductile and impact-resistant. Therefore, the A fibre envelope/B fibre core interface fails first due to the significant stress asymmetry present due to the multi-material interface. Furthermore, localized core failures act as crack nucleation sites which branch out to the interface. To summarize, the fracture of the hybrid specimens initiates in the core material (B), which has lower tensile strength, and expands to the outer synthetic envelope, which experiences complete rupture in all cases except for the B + A batch.

**Figure 9:**
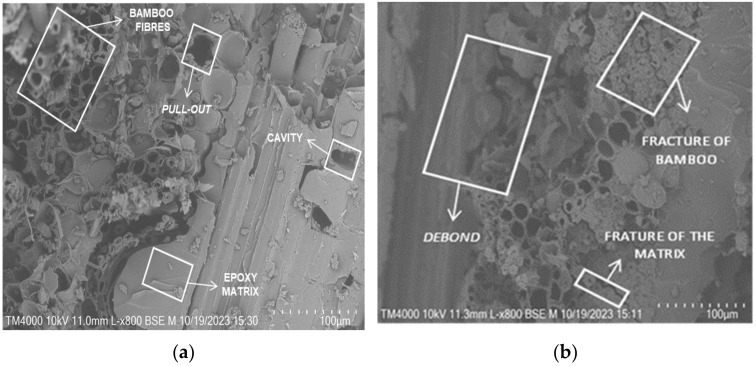

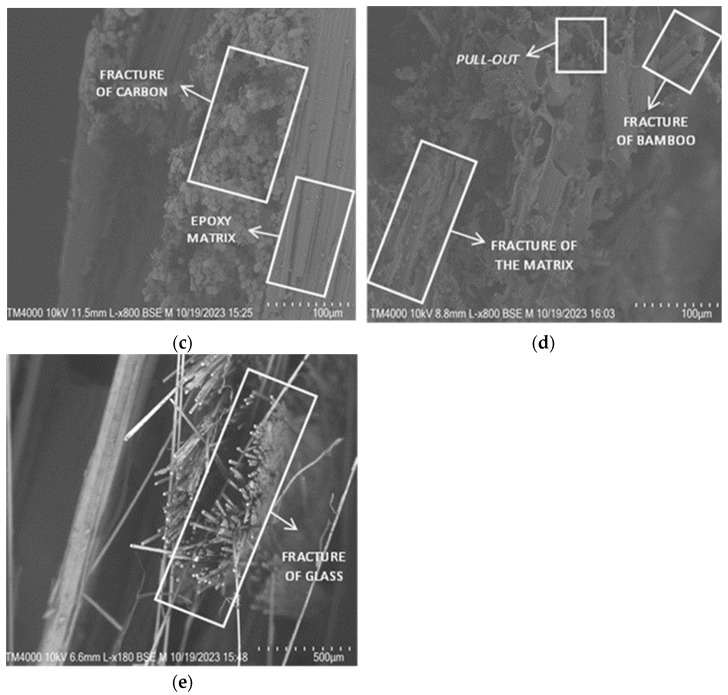
Representative SEM images of the fracture surface of tensile specimens: (**a**) B; (**b**) core B + C; (**c**) outer layer B + C; (**d**) core B + G; (**e**) outer layer B + G.

Figure 9 presents the representative SEM micrographs of the reinforced composite specimens as a function of fibre hybridization. From Figure 9a, the failure surface of the bamboo (B) specimens can be seen. A generally smooth and brittle resin failure surface can be seen as well as a debonding tendency of the bamboo fibre/resin interface. From Figure 9b, the fracture of the bamboo fibre is visible. It can be seen from the cross-section of the fibre failure that a significant variation in lumen size across the fibre bundle occurs, evidencing a large void density within the fibre, linked to lower mechanical properties of the composite material. A similar brittle resin failure can be observed for the B + C specimens. However, the carbon/resin interface is intact (see Figure 9c). From Figure 9d,e, a representative failure of the B + G specimens can be seen. A glass fibre bundle fibrillation upon failure is observed, and this is a classical failure for these fibres. Unlike the carbon bundles, a more ductile failure is evidenced and is linked to the higher ductility of the composite itself (see Table 2). In general, the failure mechanism involves a combination of matrix deformation, fibre pullout, interface filler/matrix debonding, and matrix cracking.

### 3.4. Flexural Properties

**Figure 10:**
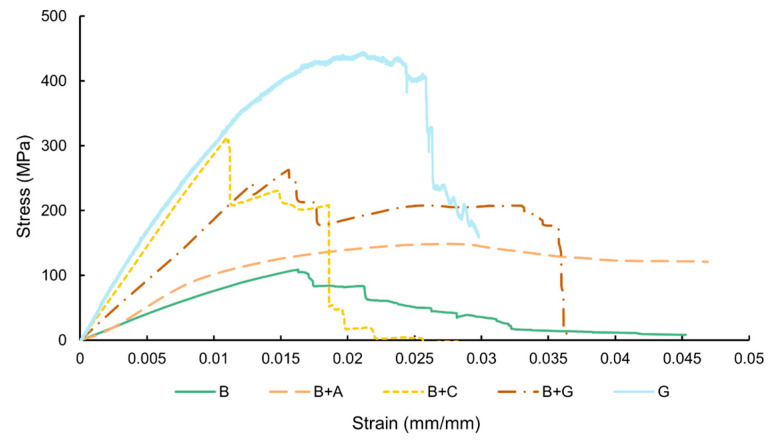
Representative flexural stress–strain curves as a function of hybridization.

**Figure 11:**
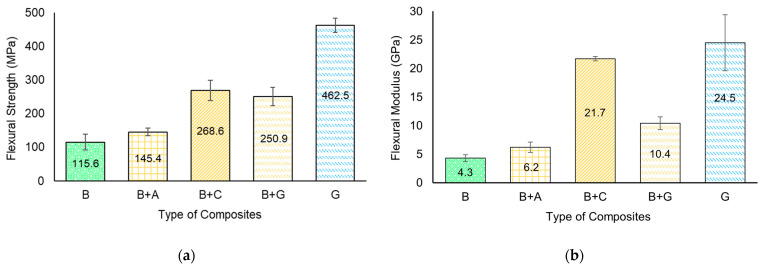
Flexural material properties as a function of hybridization: (**a**) flexural strength; (**b**) flexural modulus.

Figure 10 displays the representative flexural stress–strain curves, while Figure 11 and Table 4 present the quantitative flexural properties as a function of hybridization.

The stress–strain curve presented in Figure 10 show that the B, B + G, and B + A specimens have a higher deformation until their fracture compared to batch B + C (which can be explained by the more ductile behaviour of glass and aramid fibres compared to carbon fibre).

Similar to the tensile properties, the behaviour of the specimens up to failure varied as a function of fibre hybridization. As expected, the bamboo fibre-reinforced composite specimens presented the lowest flexural strength and stiffness. For the hybrid specimens, the strength values at failure increased as a function of the fibre type.

From Figure 11 and Table 4, it can be seen that the composites reinforced only with bamboo fibres have a flexural strength value of approx. 115.6 MPa and a flexural modulus of approx. 4.3 GPa. These results are consistent with the literature [35,37].

The integration of aramid fibre into the hybrid composites had a lower impact on flexural strength compared to the incorporation of carbon and glass fibres. This may be associated with the poor adhesion observed in the B + A batch between the external layers of aramid fibres and the bamboo core, which could have led to premature failures compromising the structural integrity of the composite, thereby reducing its flexural properties. For instance, B + A specimens presented an increase of approx. 26% when compared to the B batch composites, while B + C specimens presented an increase of approx. 132%, and B + G specimens approx. 117%, respectively. When the flexural force was applied, the external synthetic layers bore most of the applied load as the synthetic fibres had a very high load-bearing capacity compared to the bamboo fibre.

From Figure 11b and Table 4, it can be seen that, similar to flexural strength, the hybridization also caused a significant increase in the flexural modulus for all hybrid composite batches when compared to the B specimens. For instance, for B + A specimens, the flexural modulus, showed an increment by approx. 44% (from 4.3 GPa to 6.2 GPa), for B + C specimens an increase of approx. 405% (from 4.3 GPa to 21.7 GPa), while for the B + G specimens approx. 142%. (from 4.3 GPa to 10.4 GPa).

**Figure 12:**
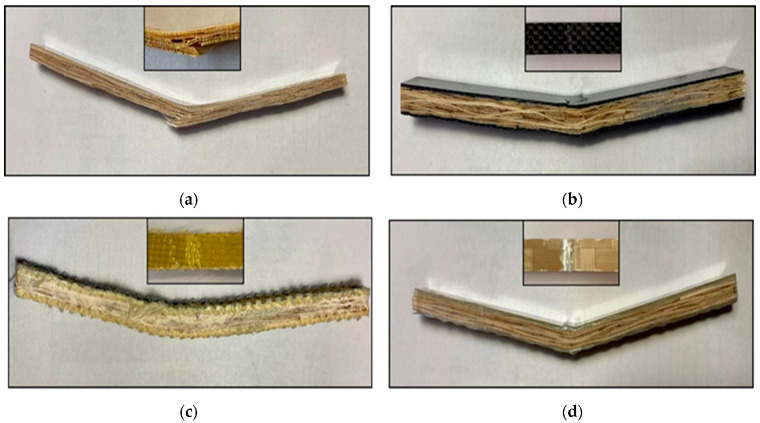
Representative flexural specimen failure mode as a function of fibre hybridization: (**a**) B; (**b**) B + C; (**c**) B + A; (**d**) B + G.

Figure 12 illustrates the typical failure modes of flexural specimens as a function of fibre hybridization. From Figure 12a, a representative failure mode can be seen for the neat bamboo specimens, and a mixed failure mode of bottom face traction and cross-sectional cracking is visible. This indicates higher compression resistance for the B composites. From Figure 12b, the representative flexural failure of the B + C case can be seen. A classical compressive failure is visible on the top face with no other significant failure zones. This is a very common failure for synthetic fibres given the very high traction resistance of the carbon fibre. For B + A specimens, a zone of fibre buckling under compression is visible on top of the specimen, similar to the previous case (see Figure 12c). Finally, for the B + G specimens (Figure 12d), it is evident that the top layers of glass fibres exhibit compression failure, characterized by visible buckling and delaminated zones (whiteish delaminated zones). These synthetic fibre buckling failures under compression are to be expected under three-point bending stresses.

### 3.5. Impact Properties

**Figure 13:**
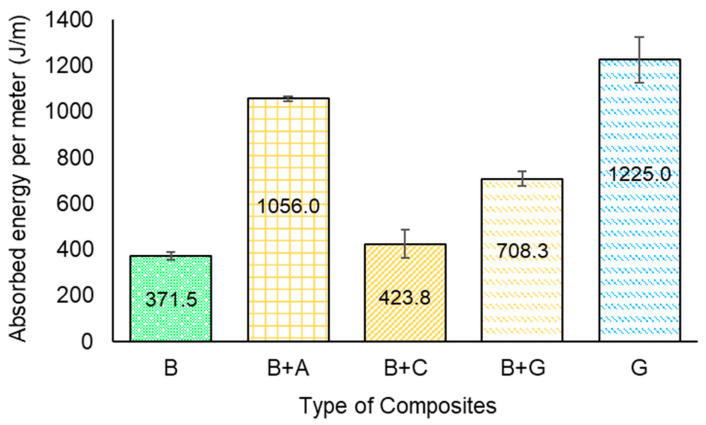
Impact energy absorption of the composites.

Figure 13 shows the impact energy absorption of the composites as a function of hybridization. It can be seen that the impact strength of the hybrid composites greatly dependent on the fibre type.

For the B specimens, an impact energy absorption value of approx. 371.5 J/m was obtained. Overall, hybridization improved the impact absorption properties of composites reinforced only with natural fibres, which was expected and followed trends in the literature [38]. The hybrid composites followed the same trends as composites reinforced with synthetic fibres, with the best results achieved for batch B + A (1056 J/m), where the addition of aramid fibres led to an increase of approx. 184% compared to batch B. For B + C and B + G specimens, increases of approx. 14% and approx. 91% were obtained, respectively. The smaller increase found for the batch B + C was associated with the brittle behaviour of carbon fibres. Finally, the values obtained for batch G were consistent with the literature [24]. It was shown in the literature that the impact failure occurs either by interlaminar delamination or fibre pullout/fibre breakage [39].

### 3.6. Thermal Properties

**Figure 14:**
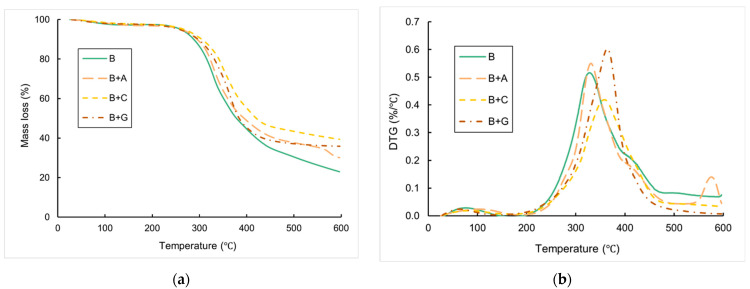
Representative TG curves of the composite specimens: (**a**) TG; (**b**) DTG analysis.

TGA was employed to examine how fibre hybridization impacts the thermal characteristics of the composites. The findings are shown in Figure 14 and summarized in Table 5. Parameters such as the initial decomposition temperature (*T*_IDT_), final decomposition temperature (*T*_FDT_), and char residue were determined from the TG curves for each case studied. These parameters serve as indicators of the thermal stability of the composites [40,41]. It is widely recognized that the thermal stability of composites is associated with the onset of significant weight loss, evident as a distinct downward trend in the TG curve, visible at 320–420 °C in Figure 14.

As anticipated, the hybridization of the composites increased the maximum degradation temperature in comparison to the pure bamboo. For instance, the composite samples presented an increment in *T*_IDT_ when compared to the B composite samples (293.7 °C for B + A samples and 302.5 °C and 311.8 °C for B + G samples compared to 286 °C of B samples). This aligns with the existing literature indicating that synthetic fibres enhance the thermal characteristics of composites [42]. Overall, B + C samples showed better results, as expected, which highlighted carbon fibre among the synthetic fibres used as having a higher degradation temperature. Finally, the hybridization reduced the rate of mass loss compared to composites reinforced solely with bamboo, and this trend is also observed in the literature [18].

From the DTG analysis (see Figure 14b), it can be observed that the curves related to composites containing natural reinforcement (bamboo) have two associated stages (except for batch B + A, which has three stages, with the last one related to the degradation of aramid fibres at around 550°C). The first stage is related to the evaporation of moisture from the fibres, while the second is due to a pyrolysis process associated with the degradation of the main constituents of bamboo fibres (cellulose, hemicellulose, and lignin) [42]. This process happens simultaneously with the degradation of the matrix, which occurs at around 300°C (according to information provided by the supplier). The behaviour presented by the curves is similar for all hybrid composites. It is worth noting batch B + G for the higher rate of mass loss concerning the temperature associated with the pyrolysis process.

To conclude, the hybridization with synthetic fibres increased the thermal properties of the bamboo fibre-reinforced composites studied here.

## 4. Conclusions

This work focused on the fabrication and morphological, physical, mechanical, and thermal testing of bamboo/synthetic fibre-reinforced hybrid composites. Neat bamboo and hybrid composites were fabricated via the vacuum bagging technique. An architecture of five layers of bamboo fabrics surrounded on each side by two layers of synthetic fabrics of bidirectional carbon/glass/aramid fabrics was used and characterized by several tests. The following conclusions can be drawn:

- The densities obtained for the composites followed the trend of the densities exhibited by the fibres, with the batch B composites having the lowest values of this parameter (0.95 g/cm^3^), followed by the B + A (0.97 g/cm^3^) and B + C (1.02 g/cm^3^) composites.
- The hybridization of bamboo fibre-reinforced composites with synthetic fibres significantly improved all composite mechanical properties, as expected. The highest improvement on the tensile strength parameter was found for batch B + A, with an improvement of approx. 111% when compared to the neat bamboo-reinforced composites. The Young’s modulus presented a significant tendency of improvement as a function of fibre type, where the B + C specimens presented the highest tensile modulus when compared to the B specimens, at approx. 150%. Overall, the tensile failure modes initiated in the core material (bamboo), which had lower tensile strength, and expanded to the outer synthetic envelope, which experienced complete rupture in all cases except for the B + A batch.
- The hybridization improved the flexural properties for all hybrid composites. The highest values of flexural strength and modulus were obtained by the incorporation of carbon fibres (an increase of approx. 132% in flexural strength and approx.405% of flexural modulus was found for the B + C specimens when compared to the B specimens).
- All the hybrid composites presented enhancements in impact energy when compared to the neat bamboo fibre-reinforced composites. The best results for impact were obtained for batch B + A (1.06 kJ/m), as expected, due to the excellent impact properties of aramid fibres, with an enhancement of approx. 184% compared with the B specimens.
- The hybridization with synthetic fibres increased the thermal properties of the bamboo fibre-reinforced composites studied. The hybridization reduced the rate of mass loss compared to composites reinforced solely with bamboo.

In summary, the use of bamboo fibre composites enveloped by synthetic fibres could provide a range of benefits to various industrial sectors, especially the automotive industry. These composites may contribute to enhanced durability, weight reduction, cost-efficiency, and design flexibility while providing protection against environmental factors and contributing to improved environmental performance in the automotive sector. Future research efforts will focus on the development of new sustainable materials using bio-based resins derived from plant sources in conjunction with natural fibres to enhance the sustainability of composites. This approach not only reduces reliance on petroleum-based resins but also will contribute to a more circular economy by offering recyclable materials.

## Figures and Tables

**Table 1:** Basic parameters of the fabrics used.

Woven Style	Bamboo Fabric	Glass Fabric	Aramid Fabric	Carbon Fabric
Woven style	2 × 2 Twill	Plain	Plain	Plain
Density (g/cm^3^)	0.7–1.2	2.5	1.4	1.8
Weight (g/m^2^)	450	500	300	196
Thickness (mm)	0.80	0.65	0.40	0.25
Orientation (angle)	0/90°	0/90°	0/90°	0/90°

**Table 2:** Geometric and apparent densities of the composites studied.

Batch of Specimens	Geometric Density (g/cm^3^)	Apparent Density (g/cm^3^)
B	0.95 ± 0.20	1.00 ± 0.04
B + A	0.97 ± 0.24	0.98 ± 0.07
B + C	1.02 ± 0.18	1.10 ± 0.05
B + G	1.24 ± 0.29	1.26 ± 0.02
G	1.85 ± 0.28	1.91 ± 0.06

**Table 3:** Quantitative tensile test data as a function of hybridization.

Batch of Specimens	Tensile Strength (MPa)	Young’s Modulus (GPa)	Strain (%)
B	64.9 ± 6.7	6.3 ± 0.61	0.99 ± 0.02
B + A	136.6 ± 8.4	11.4 ± 3.1	1.33 ± 0.42
B + C	124.3 ± 15.8	15.8 ± 2.1	0.66 ± 0.079
B + G	114.9 ± 10.1	10.1 ± 1.2	1.00 ± 0.12
G	306.8 ± 14.2	21.9 ± 0.7	1.14 ± 0.34

**Table 4:** Quantitative flexural test data.

Batch of Specimens	Flexural Strength (MPa)	Flexural Modulus (GPa)	Strain (%)
B	115.6 ± 23.6	4.3 ± 0.6	10.41 ± 8.19
B + A	145.4 ± 11.2	6.2 ± 0.9	5.96 ± 1.47
B + C	268.6 ± 30.5	21.7 ± 0.4	2.87 ± 0.20
B + G	250.9 ± 27.7	10.4 ± 1.1	5.34 ± 1.64
G	462.5 ± 21.3	24.5 ± 4.9	2.74 ± 0.14

**Table 5:** Thermal analysis results.

Composite	*T* _(100 °C)_ (%)	*T*_IDT_ (°C)	*T*_FDT_ (°C)	Residual Mass (%)
B	2.24	286	377	22.89
B + A	1.74	293.7	386.3	30.10
B + C	1.51	302.5	412.9	39.37
B + G	1.96	311.8	397.5	35.85

## Data Availability

Data are contained within the article.
